# Defect-assisted synthesis of magneto-plasmonic silver-spinel ferrite heterostructures in a flower-like architecture

**DOI:** 10.1038/s41598-020-73502-5

**Published:** 2020-10-12

**Authors:** Marco Sanna Angotzi, Valentina Mameli, Claudio Cara, Vincenzo Grillo, Stefano Enzo, Anna Musinu, Carla Cannas

**Affiliations:** 1 Department of Chemical and Geological Sciences, University of Cagliari, S.S. 554 Bivio per Sestu, 09042 Monserrato, Italy; 2 Consorzio Interuniversitario Nazionale Per La Scienza e Tecnologia Dei Materiali (INSTM), Via Giuseppe Giusti 9, 50121 Florence, Italy; 3 Istituto Nanoscienze Consiglio Nazionale delle Ricerche (CNR-NANO), Via G. Campi 213/a, 41125 Modena, Italy; 4 Department of Chemistry and Pharmacy, University of Sassari, Via Vienna 2, 07100 Sassari, Italy

**Keywords:** Materials chemistry, Physical chemistry, Chemical synthesis, Chemistry, Materials science, Nanoscience and technology

## Abstract

Artificial nano-heterostructures (NHs) with controlled morphology, obtained by combining two or more components in several possible architectures, make them suitable for a wide range of applications. Here, we propose an oleate-based solvothermal approach to design silver-spinel ferrite flower-like NHs. Small oleate-coated silver nanoparticles were used as seeds for the growth of magnetic spinel ferrite (cobalt ferrite and spinel iron oxide) nanodomains on their surface. With the aim of producing homogeneous flower-like heterostructures, a careful study of the effect of the concentration of precursors, the reaction temperature, the presence of water, and the chemical nature of the spinel ferrite was carried out. The magnetic and optical properties of the NHs were also investigated. A heterogeneous growth of the spinel ferrite phase on the silver nanoparticles, through a possible defect-assisted mechanism, was suggested in the light of the high concentration of stacking faults (intrinsic and twins) in the silver seeds, revealed by Rietveld refinement of powder X-ray diffraction patterns and High-Resolution electron microscopy.

## Introduction

Spinel ferrite-based nanoheterostructures (NHs) have attracted considerable interest in the last decades, thanks to the possibility of joining in a single material magnetic and other physical–chemical properties. Indeed, spinel ferrites represent a class of ferrimagnetic materials widely studied thanks to the chemical and mechanical stability, and the possibility to tune the hard or soft magnetic behaviour by changing the type of the divalent cation^1–6^. Furthermore, noble metals (*e.g.*, Ag, Au) have numerous properties (optic, catalytic, antibacterial) that find application in several fields^7^. Between them, silver presents several advantages in terms of cost, availability, but also activity as antimicrobial^8^. Silver-ferrite NHs are used, for example, as substrates for surface-enhanced Raman spectroscopy (SERS), thanks to the high surface resonance effect of silver^9–11^. Electromagnetic enhancement, caused by the construction of “hot-spots” in aggregated NHs, can contribute to optimizing the SERS performances. In this context, magnetic-silver NHs represent an excellent material for SERS activity, since spinel ferrite nanoparticles can induce magnetic aggregation. Again, these kinds of NHs are employed as catalysts for purification of dye effluents^11,12^, exploiting the catalytic activities of silver, and the magnetic separation of spinel ferrite. Flower-like silver-magnetite heterostructures have been employed as an antibacterial against Escherichia coli^12,13^. Moreover, silver-spinel ferrite NHs have been utilized for combined photothermal and magnetic heating, exploiting the localized surface plasmon resonance effect of the noble metal part and the magnetic behavior of the spinel ferrite^14–16^.

The most crucial and challenging aspect in designing heterostructures is the development of synthesis methodologies able to produce highly crystalline particles having low size dispersity, tuneable size, and shape, and defined interfaces^17^. In the literature, silver-spinel ferrite NHs have been synthesized in forms of many architectures, from core-shell^18,19^ to dimers^18–24^ and flower-like^9–11,25–28^, prepared via one-pot^9,10,28^ or two-pot syntheses (Table 1S)^9–11,18–28^. For example, Fe_3_O_4_@Ag flower-like NHs have been synthesized by a seed-mediated growth approach in organic solvents starting from magnetite NPs with silver nitrate or silver oleate as Ag precursors^25^. Dimer Ag-ferrite NHs have also been synthesized by various authors^20–24^, through thermal decomposition of iron acetylacetonates or oleates in the presence of silver NPs (seed-mediated growth). Other less conventional approaches concern the surface reduction of silver nitrate in the presence of a reducing agent (*e.g.*, glucose, oleylamine, etc*.*) on spinel ferrite NPs to produce dimer and flower-like NHs^11,26,27^, or surface oxidation of Ag@Fe core–shell NPs in the air for the production of core–shell (or flower-like) NHs having a silver core and magnetite/maghemite shell^18,19^. Most of the above-cited syntheses are afforded through the well-known surfactants-assisted high-temperature decomposition of organic complexes in high boiling organic solvents. Nevertheless, the necessity of more eco-friendly strategies that use less amount of toxic organic solvents, less expensive precursors, and lower temperatures, has moved the interest toward alternative synthetic approaches^29^. In this context, the solvothermal methods feature several advantages such as the use of low-boiling and inexpensive solvents, the ease of the synthesis, lower temperature and the possibility to monitor it together with the pressure^30–34^.

Recently, an oleate-based solvothermal method has been employed for the synthesis of spinel ferrite-based core–shell NHs with good control over crystallinity, size, shell thickness, and interface^6,35^. Another important advantage deriving from the above-cited method lies in the central role of the biocompatible oleate molecule, acting as the precursor, the surfactant, and the capping agent, and avoiding the employment of other chemicals and the related compatibility issue. In this work, this oleate-based solvothermal method was employed to prepare silver-spinel ferrite NHs having flower-like architecture. The seed-mediated growth approach was exploited starting from silver seeds, and several synthetic parameters were studied, namely (i) the metal oleate concentration, (ii) the reaction temperature tested with (iii) different amount of solvents, (iv) the presence of water, (v) and the chemical nature of the metal oleate. Structural and morphological properties were studied through powder X-ray diffraction (XRD), conventional and high-resolution transmission electron microscopy (TEM/HRTEM). The optical and magnetic properties of the silver and spinel ferrite parts of the heterostructures were instead analyzed utilizing UV–Vis spectroscopy, DC magnetometry, and ^57^Fe Mössbauer spectroscopy.

## Results and discussion

### Synthesis of the silver seeds

**Figure 1:**
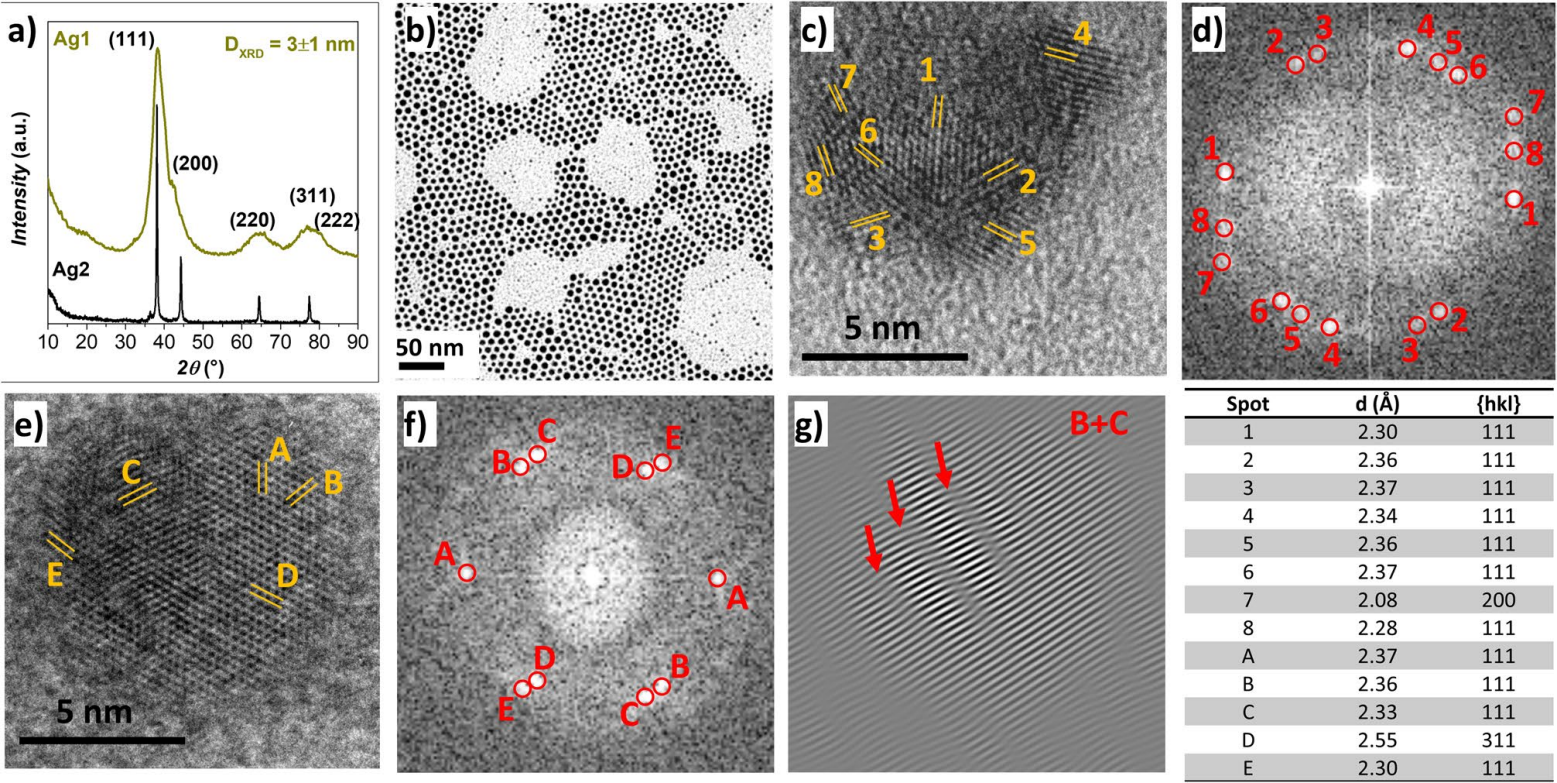
XRD pattern of Ag1 and Ag2 samples (**a**), TEM image (**b**), HRTEM images (**c**, **e**), FFT (**d**, **f**), masked inversed FFT (**g**), interplanar indexes of the Ag1 sample obtained by Digital Micrograph.

Silver oleate, insoluble in most organic solvents, is soluble in oleic acid at 100 °C, and hence oleic acid can be used as the solvent, capping, and reducing agent^36^. Indeed, at high temperature (150–170 °C) the pale-yellow solution became brown and then dark purple due to the formation of NPs through thermal reduction of silver oleate by oxidation of the double bond of oleic acid^36^. The reaction must be conducted under an inert atmosphere to prevent the oxidation of oleic acid by oxygen. The presence of the capping molecules on the NPs’ surface was confirmed by FTIR and TGA (Fig. 1S), revealing the typical bands (CH and COO^-^ stretching modes)^37^ and the decomposition temperature (260 °C)^30,38^ of the oleate molecules. This green synthesis, proposed by Xu et al.^36^ thanks to the high quantity of capping agent, allows to obtain very small NPs, as evidenced from XRD, TEM, and HRTEM measurements in Fig. 1.

**Figure 2:**
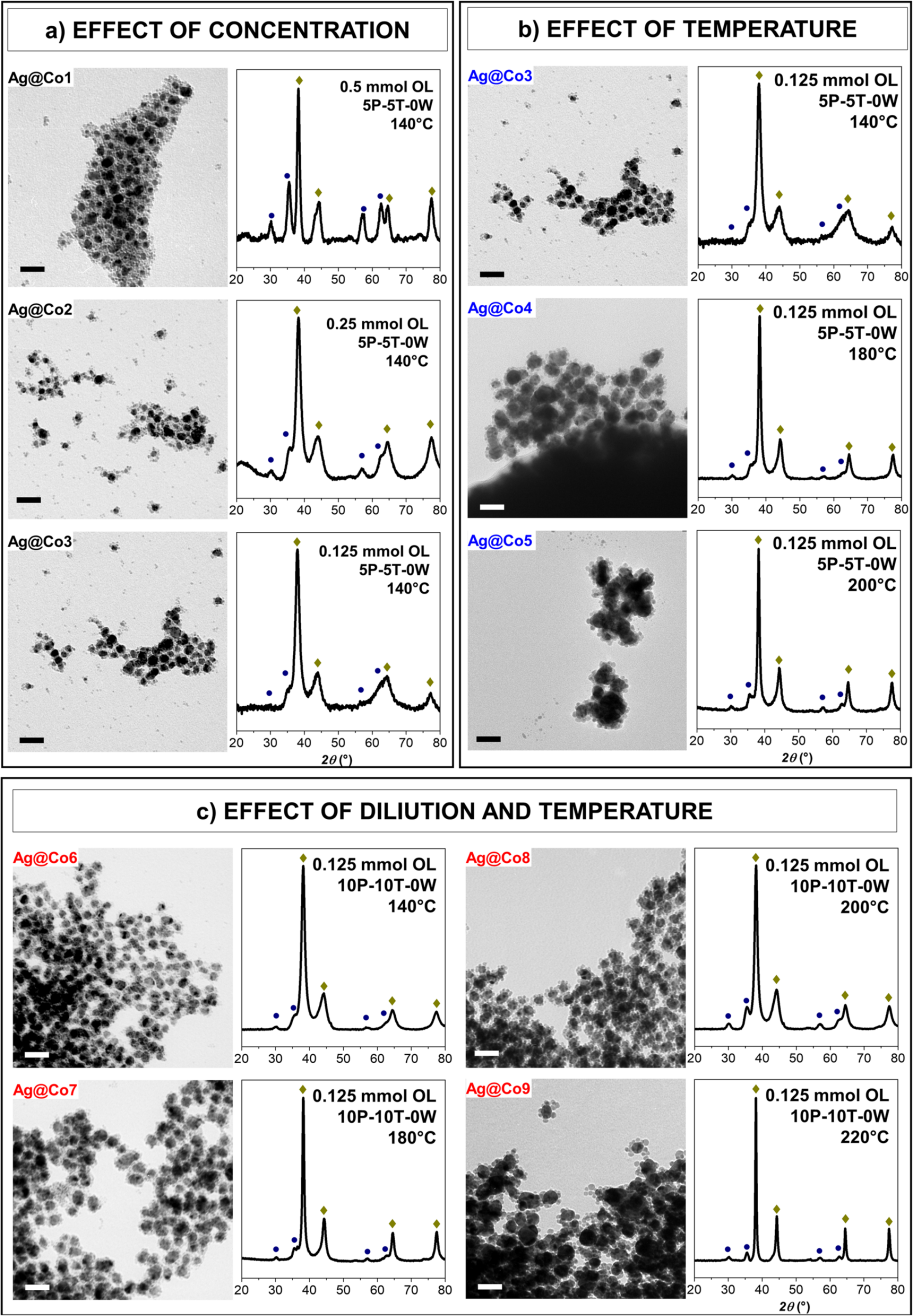
Effect of concentration (**a**), temperature (**b**), and dilution and temperature (**c**) for the silver-spinel ferrite heterostructure: TEM bright-field images are shown in the left part; the scale bar is 50 nm. XRD patterns are shown in the right part; the golden rhombus indicates silver reflexes; the blue circle indicates spinel ferrite reflexes.

**Figure 3:**
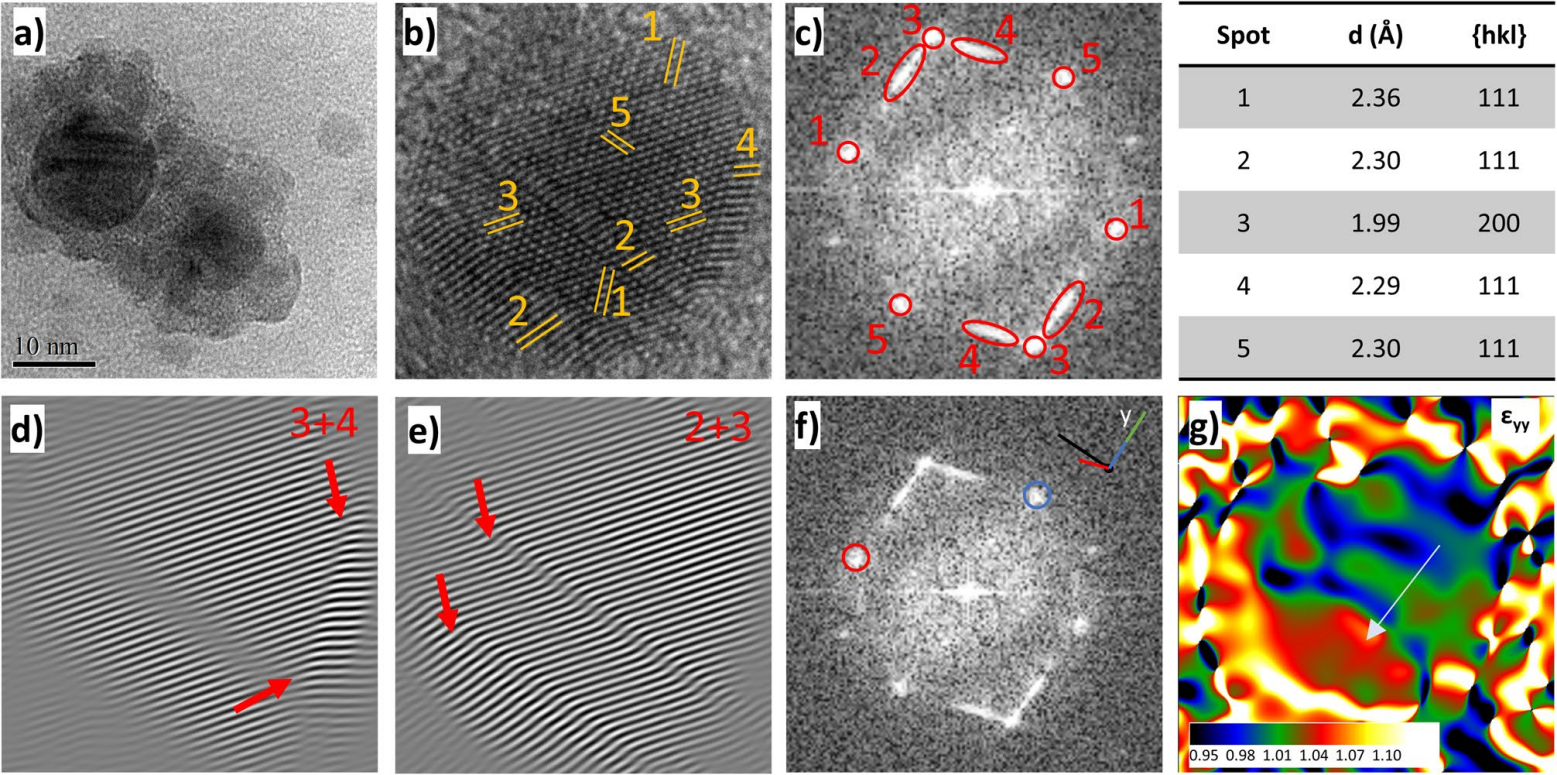
TEM (**a**) and HRTEM (**b**) images, FFT (**c**, **f**), masked inversed FFT (**d**, **e**) obtained by Digital Micrograph, and GPA analysis obtained with STEM_CELL^51,52^ (**g**) of the sample Ag@Co1.

The crystallite size estimated from single peak analysis (Scherrer equation) was about 3 nm, while D_TEM_V_ was 7 ± 1 nm. The crystalline phase of the sample Ag1 consists of cubic silver (fcc), but some peculiarities can be noted. Indeed, it is possible to observe how the {200} peak is shifted toward lower angles (~ 2°), getting close to the {111} (Fig. 1), which is also slightly shifted at higher angles (0.5°) and is sharper than all the other peaks (Fig. 2S). It is also important to observe that all the peaks are symmetric. This behavior could be ascribed to the presence of planar defects that may cause a partial transition from fcc to hcp silver structure^39–41^. The theory for stacking faults interpretation was firstly developed by Warren^42^, who described as the intrinsic (α’), extrinsic (α”), and twin faults (β) may affect the line profile, in terms of shift, broadening, and asymmetry (Fig. 3S, Table 3S). This model has been subsequently revised and updated^43–45^, but it still represents a valid and effective method for the estimation of planar defects^46,47^.

Our results, in terms of peak broadening and shift, are consistent with Warren’s predictions revised by Wagner^48^. Indeed, the ratio between the crystallite sizes calculated for the {111} and {200} is 1.8, consistent with the theoretical one (1.7–2.0). Moreover, the most intense shift of the {200} peak toward lower angles agrees with the j $\stackrel{-}{G}$ values hypothesized by Wagner that are indications of the intensity and direction of the peak shift (Table 2S).

**Figure 4:**
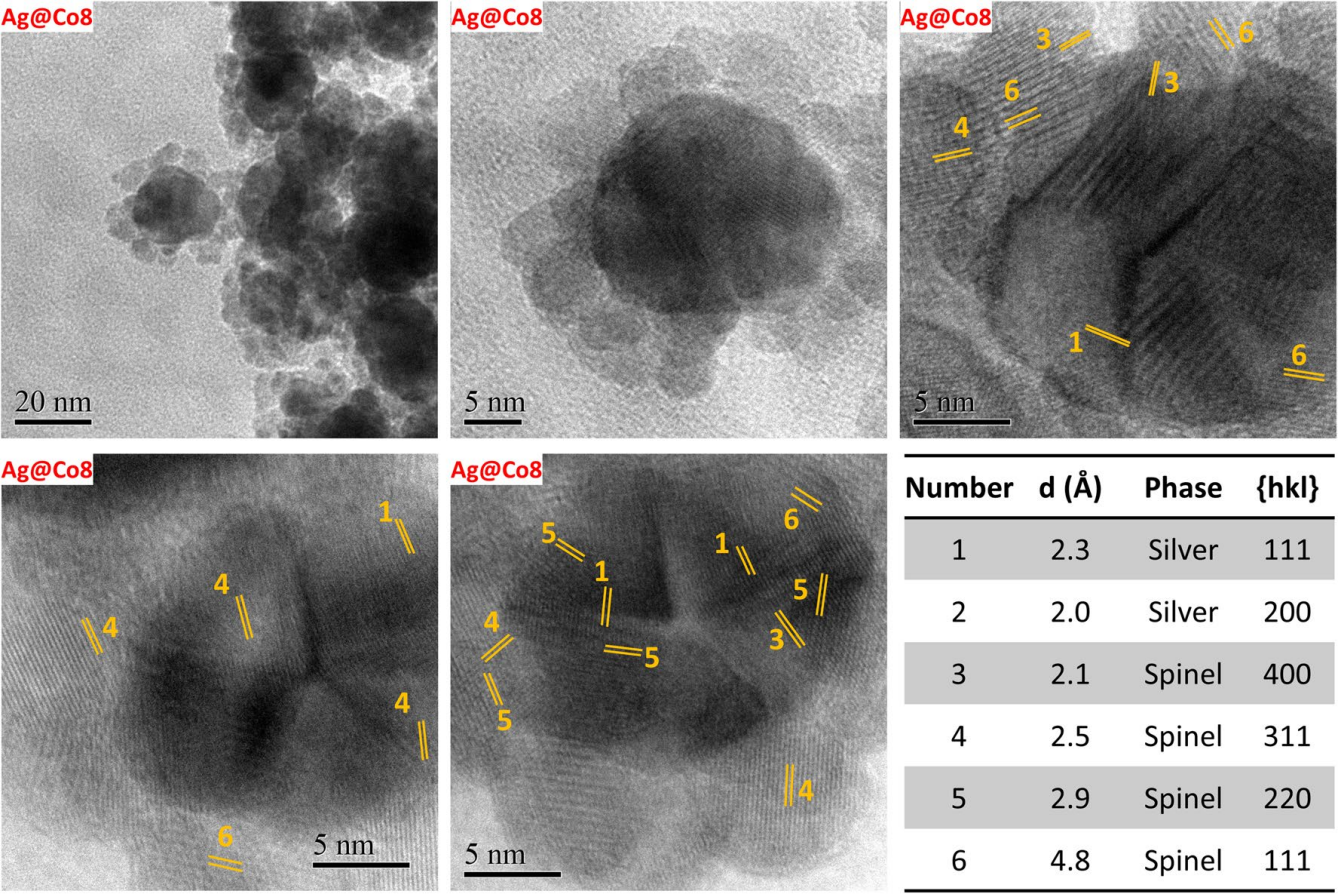
HRTEM images and interplanar indexes of the sample Ag@Co8 obtained by Digital Micrograph.

Rietveld analysis was therefore performed, by employing several adjustments, to deepen the stacking faults nature (Fig. 4S, Table 3S). At first, only silver fcc structure was adopted, giving an R_wp_ value of 9.3%, a clear mismatching in the positions and intensity of the {111} and {200} peaks, with some deviations of the fit at high angles. When both fcc (ABCABC sequence) and hcp (ABABAB sequence) structures of silver were imposed, the refinement gave better results (R_wp_ 5.4%). Finally, a refinement of the sole fcc structure with the Warren planar defects model was tested, for which a 3.6% R_wp_ was obtained, mainly due to a better refinement in the range encompassing the {111} and {200} peaks, suggesting that the planar defects are principally located in these planes of the fcc structure. Moreover, from the Rietveld refinement, it is possible to evidence that, among the stacking faults, the intrinsic (α’, 0.14) are more prominent than the extrinsic (α”, 7·10^–3^) and twin faults (β, 4·10^–4^), indicating the absence of planes in the ABCABC sequence (Table 3S). Furthermore, the lowest value found for the twin faults justifies the absence of significative asymmetry of all the peak shape (Fig. 2S), as stated by Wagner^48^. Considering that the refinement using both fcc and hcp structures did not give better results if compared to the only fcc with Warren correction, it is possible to conclude that the most probable missing planes are A or B rather than C.

HRTEM images show the planes associated with the fcc silver structure and several twinned crystals between the {111} planes, e.g., 6–2, 3–5, B-C reported in Fig. 1 d, f, and g. All the above findings permit us to conclude about the presence of stacking faults, especially intrinsic and twin faults, in the silver nanoparticles.

**Figure 5:**
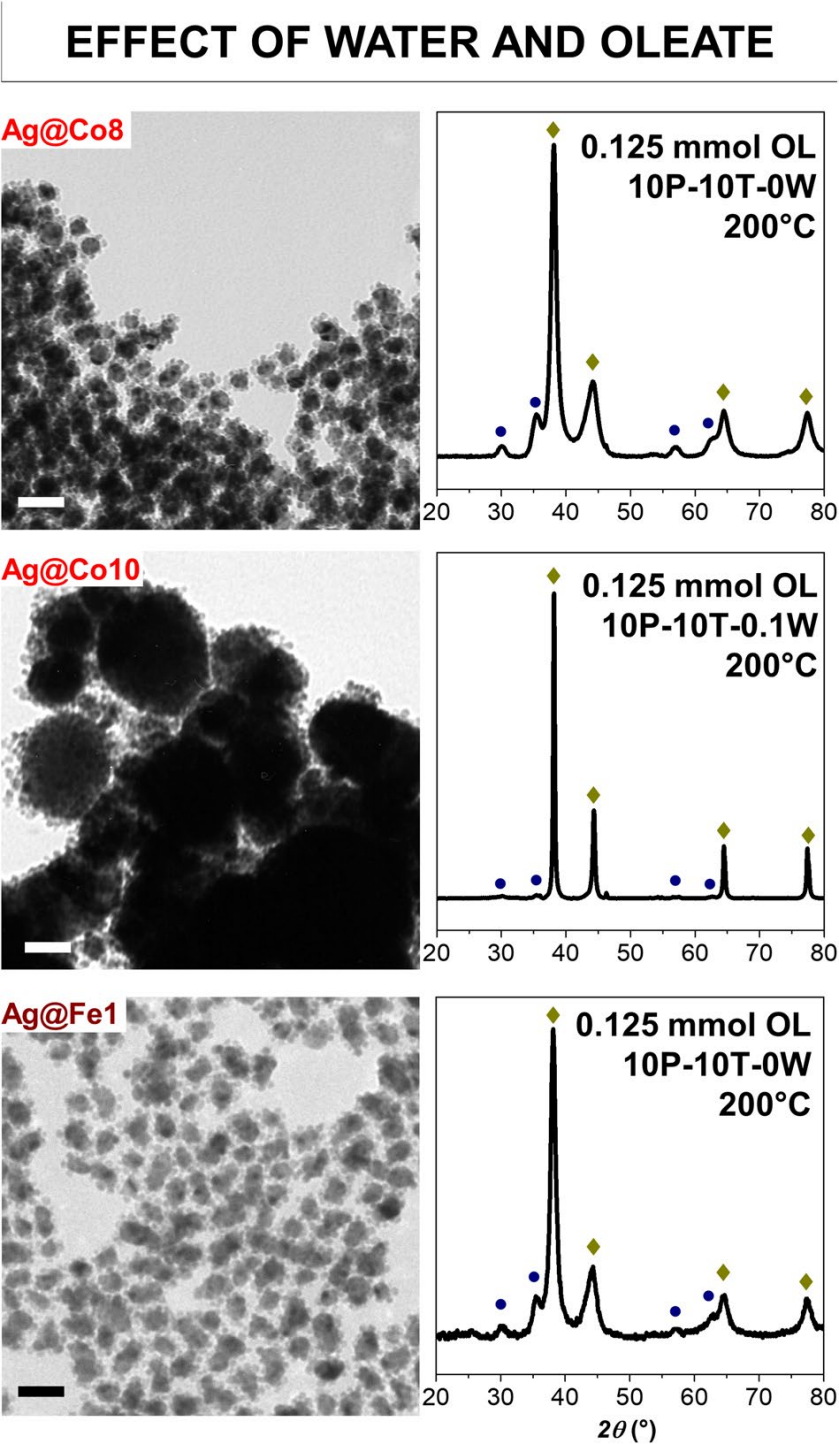
Effect of water (Ag@Co10) and chemical nature of the oleate (Ag@Fe1) in comparison with Ag@Co9: TEM bright-field images are shown in the left part; the scale bar is 50 nm. XRD patterns are shown in the right part; the golden rhombus indicates silver reflexes; the blue circle indicates spinel ferrite reflexes.

It is now interesting to compare the sample Ag1 with the sample Ag2, that was obtained by treating the sample Ag1 with a solvothermal treatment in the absence of metal oleate (Fig. 1). The XRD pattern features sharper peaks, as expected, due to the increased crystallite size (over 40 nm), having their position in close agreement with the pure fcc structure of silver, with no shifts, asymmetries, or broadening of the peaks in the all angle range. Indeed, the pattern could be refined satisfactorily with the fcc structure and without Warren corrections or hcp contributions (Fig. 5S, Table 3S). The absence of structural defects and the large size of Ag2 indicate the occurrence of Ostwald ripening phenomena, due to the reduced size and the presence of a high concentration of defects in the starting Ag1 nanoparticles, that led to the dissolution of the smaller particles and the growth of the larger ones with complete suppression of the defects.

### Synthesis of silver-spinel ferrite heterostructures

Silver-ferrite heterostructures were prepared starting from oleate-capped Ag1 NPs as seeds and metal oleate (Co^II^-Fe^III^) as ferrite precursor. Since both components are dispersible/soluble in the same media, the homogeneity of the synthesis should ensure an ideal starting condition. Several aspects were tested to find the best synthetic conditions to prepare homogeneous silver-spinel ferrite heterostructures. XRD and TEM analyses were carried out, and the results are summarized in Table 1.

**Table 1:** Synthesis conditions and features of silver-based spinel ferrite heterostructures. The reaction time was 10 h, the silver seeds were 0.2 mmol. Ol: metal oleate; P: 1-pentanol; T: toluene; W: water; T: temperature. < ε > : microstrain; a: cell parameter; %w/w: weight percentage calculated by Rietveld refinement; < D_XRD_ > : crystallite size; < D_TEM_V_ > : volume-weighted particle size calculated from TEM images.

Sample	Ol (mmol)	P (mL)	T (mL)	W (mL)	T (°C)	Phase	a (Å)	% w/w	< ε >	< D_XRD_ > (nm)	< D_TEM_V_ > (nm)
Ag@Co1	0.5	5	5	0	140	CoFe_2_O_4_	8.413	76	1∙10^-6^	7(1)	7(1)
						Ag FCC	4.089	24	2∙10^-3^	19(2)	20(5)
Ag@Co2	0.25	5	5	0	140	CoFe_2_O_4_	8.450	48	8∙10^-3^	6(1)	5(1)
						Ag FCC	4.100	52	6∙10^-7^	15(1)	15(4)
Ag@Co3	0.125	5	5	0	140	CoFe_2_O_4_	8.436	25	5∙10^-3^	8(1)	6(1)
						Ag FCC	4.090	75	1∙10^-5^	20(1)	18(5)
Ag@Co4	0.125	5	5	0	180	CoFe_2_O_4_	8.405	28	4∙10^-3^	9(1)	7(1)
						Ag FCC	4.089	72	2∙10^-5^	26(1)	25(2)
Ag@Co5	0.125	5	5	0	200	CoFe_2_O_4_	8.390	24	4∙10^-3^	12(1)	10(2)
						Ag FCC	4.089	76	1∙10^-4^	28(2)	31(6)
Ag@Co6	0.125	10	10	0	140	CoFe_2_O_4_	8.470	25	2∙10^-3^	6(1)	5(1)
						Ag FCC	4.096	75	6∙10^-8^	22(2)	18(3)
Ag@Co7	0.125	10	10	0	180	CoFe_2_O_4_	8.437	30	5∙10^-3^	9(1)	6(1)
						Ag FCC	4.092	70	7∙10^-6^	24(2)	23(5)
Ag@Co8	0.125	10	10	0	200	CoFe_2_O_4_	8.418	34	6∙10^-3^	11(1)	7(1)
						Ag FCC	4.091	66	1∙10^-5^	29(2)	20(3)
Ag@Co9	0.125	10	10	0	220	CoFe_2_O_4_	8.391	42	3∙10^-3^	10(1)	9(1)
						Ag FCC	4.089	58	1∙10^-4^	80(10)	30(7)
Ag@Co10	0.125	10	10	0.1	200	CoFe_2_O_4_	8.396	41	7∙10^-5^	6(1)	10(2)
						Ag FCC	4.089	59	3∙10^-6^	> 100	100(20)
Ag@Fe1	0.125	10	10	0	200	Fe_3_O_4_	8.397	32	2∙10^-3^	10(1)	7(1)
						Ag FCC	4.092	68	1∙10^-6^	20(2)	21(2)

### Effect of the magnetic precursor concentration

As a first attempt, following similar molar ratios already tested for spinel ferrite-based core–shell NPs^6,35^, 25 mg of Ag1 NPs, 0.5 mmol of mixed Co-Fe oleate in a mixture of 5 mL of toluene and 5 mL of 1-pentanol, were subjected to a solvothermal treatment at 140 °C for 10 h (Ag@Co1).

Even though in previous works^30,35,49,50^ water was employed as hydrolysis agent of the metal oleates, in this work, it was removed to avoid the overgrowth of silver, considering that the presence of water as an impurity in the organic solvents should assure the formation of spinel ferrite NPs. Indeed, toluene and pentanol contain 0.02% and 0.5% of water, corresponding to 1.4 mmol and 2.9 mmol when 10 mL or 20 mL of solvents in total are used, respectively (5 mL P + 5 mL T, or 10 mL P + 10 mL T). The XRD pattern (Fig. 2a, Ag@Co1) reveals the presence of peaks typical of silver in fcc structure and a spinel ferrite phase, whose weight fraction, calculated by Rietveld refinement, is 24% and 76% w/w, respectively.

**Figure 6:**
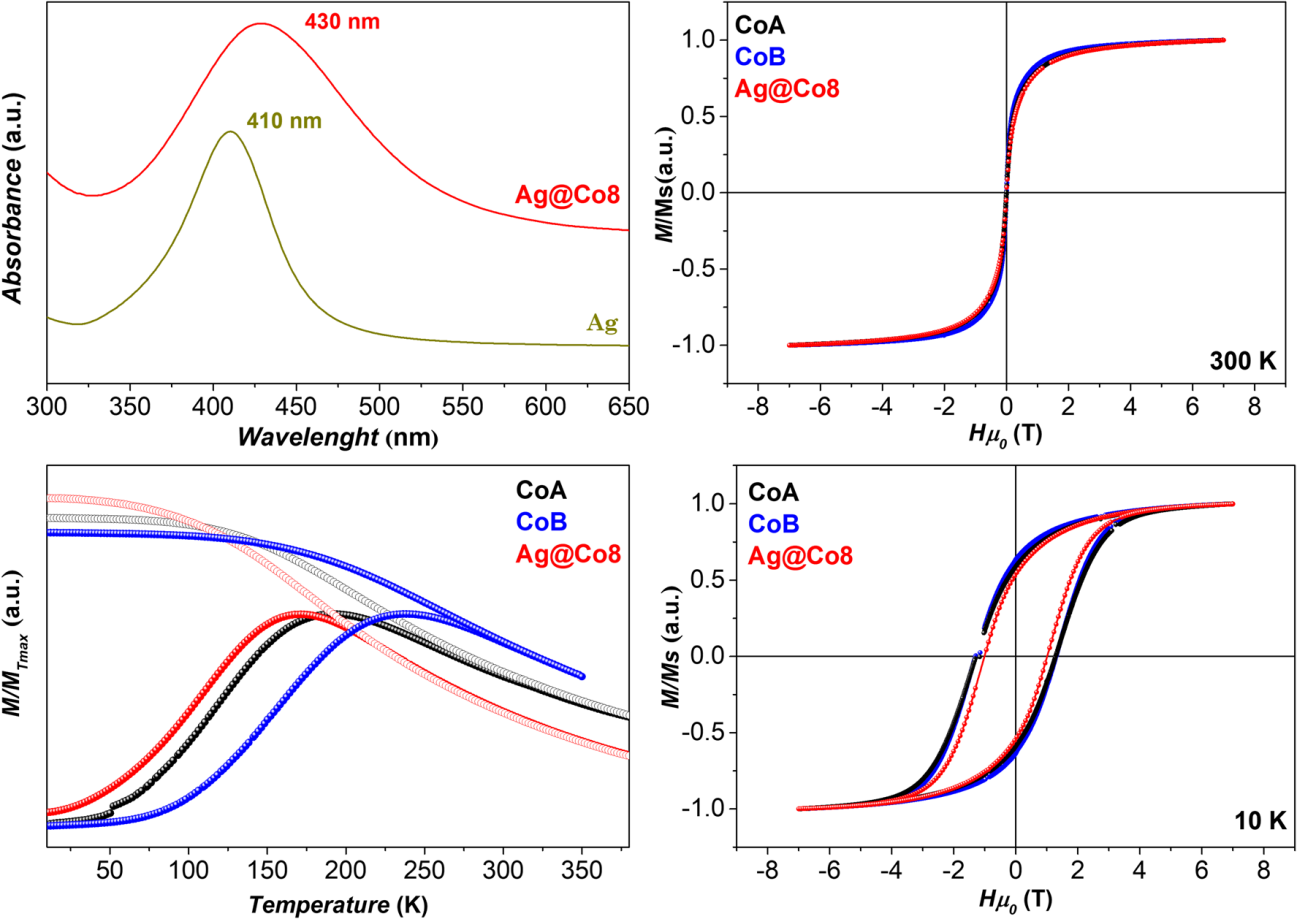
UV–Vis spectra (top left); ZFC (full circles) and FC (empty circles) curves recorded at low external magnetic field (10 mT) (bottom left); magnetization isotherms at 300 K (top right) and 10 K (bottom right)) of the heterostructures (Ag@Co8) and reference samples of oleate-capped cobalt ferrite nanoparticles of 6–8 nm in size.

**Figure 7:**
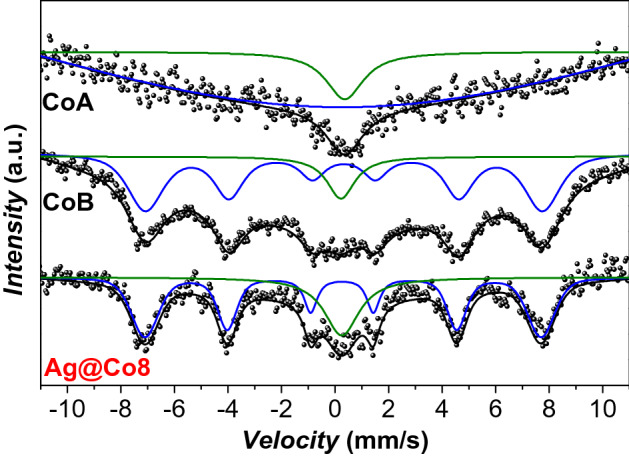
Room temperature ^57^Fe Mössbauer spectra of the samples Ag@Co8, CoA, and CoB.

The pattern was refined both in the absence and in the presence of planar defects (Table 3S, Fig. 6S). Due to the lower value of R_wp_ (3.1% vs. 3.9%), and the better definition of the {200} reflex, it can be supposed that the planar defects, especially the intrinsic stacking faults (α’, Table 3S) were also retained in the silver particles of the heterostructures. The two refinements also differ for the quantitative and size estimation of silver, being both lower when the Warren correction was not considered. Therefore, the Rietveld refinement with Warren correction was adopted for all the heterostructures, due to the better results obtained (Table 3S, Fig. 7S).

TEM and HRTEM images of Ag@Co1 (Figs. 2a, 3), show the obtainment of the flower-like silver-ferrite NHs featuring a dark silver core (20 nm) and small bright cobalt ferrite domains all around (7 nm). However, a fraction of isolated bare CoFe_2_O_4_ nanocrystals were formed. HRTEM images (Fig. 3) also reveals how silver NPs are affected by structural defects, corroborating the scenario depicted from the Rietveld analysis of the XRD patterns.

Indeed, the HRTEM image, reported in Fig. 3b, displays twin planes between the 3–2 and 3–4 lattice distances. Moreover, the 2 and 4 spots of the FFT image (Fig. 3c), corresponding to the {111} planes, show the typical elongated shape of twin planes, and the masked inversed FFT images highlight these findings, as can be seen by the red arrows in Fig. 3d and f. The geometric phase analysis (GPA, Fig. 3g) emphasizes the twinned planes, revealing an increase of lattice distance of 7% in the ε_yy_ component (strain along the y component) in the direction of the arrow, in agreement with the conversion of {200} to {111} planes (projected on the direction orthogonal to the twin plane).

Similarly to the sample Ag2 and for the same reasons, Ostwald ripening phenomena occur in the Ag1 nanoparticles during the formation of the heterostructure, as indicated by the increased particle size (from 7 to 20 nm). However, in this case, the growth of silver nanoparticles is limited, as well as the suppression of the stacking faults and twins (Table 3S), probably because of the presence of the metal oleates used as precursors of the spinel phase, acting as growth inhibitors. Taking into account that structural defects are known to be the most reactive sites, their presence on the Ag1 surface may have driven the growth of the spinel ferrite petals, suggesting a defect-assisted mechanism for the heterostructure formation^53^.

To lower the fraction of free cobalt ferrite in the heterostructures, the quantity of Co-Fe oleate was progressively decreased from 0.5 to 0.25 and 0.125 mmol (Ag@Co1, Ag@Co2, and Ag@Co3, respectively), keeping the other parameters unchanged (Fig. 2a). Both silver and spinel ferrite sizes did not change significantly with the concentration (Table 1). The relative fraction of cobalt ferrite decreased from 76% w/w to 25% w/w, as also evident from TEM images (Fig. 2a), where it is possible to observe the progressive reduction of the number of the isolated cobalt ferrite NPs. The hampering of homogeneous nucleation in favor of the heterogeneous one by controlling the ratio Ag/M-Oleate has also been observed in the literature for the synthesis of gold-spinel ferrite NHs by thermal decomposition of iron acetylacetonate on Au seeds^54^, but never studied for the reactants and methods used in this work.

### Effect of the temperature

Keeping constant the amount of metal oleate (0.125 mmol), a further step was the increase of the reaction temperature, from 140 °C (Ag@Co3) to 180 °C (Ag@Co4) and 200 °C (Ag@Co5) (Fig. 2b). The temperature influenced the size of the two phases: from 20 to 28 nm for the D_XRD_, and from 18 to 31 nm for the D_TEM_V_ of silver; from 8 to 12 nm for the D_XRD_, and from 6 to 10 nm for the D_TEM_V_ of the spinel ferrite. However, the silver and cobalt ferrite relative fractions remained almost unchanged. TEM images also showed a significant decrease in the number of free cobalt ferrite nanoparticles, indicating that the high temperature favors the formation of the heterostructure, as also evidenced for gold-spinel ferrite systems^54,55^.

### Effect of the dilution and temperature

To avoid the increase in the size of the crystalline domains, a halving of the concentration of the reactants by doubling the solvent content (20 mL in total instead of 10 mL) was tested, as reported in Fig. 2c.

In detail, Ag@Co6 was prepared as Ag@Co3 (Fig. 2b), at 140 °C with 0.125 mmol of metal oleate, but in the presence of 10 mL of pentanol and 10 mL of toluene (20 mL in total) instead of 5 mL (10 mL in total). The two samples featured similar sizes (Table 1), but TEM images revealed a decrease in the number of free ferrite NPs in favor of more homogeneous silver-ferrite heterostructures. In this promising conditions, the temperature effect was also tested at double solvent content (20 mL in total), starting from Ag@Co6, prepared at 140 °C, and increasing the temperature to 180, 200, and 220 °C, for Ag@Co7, Ag@Co8, and Ag@Co9, respectively (Fig. 2c). The size of the spinel ferrite part slightly grew from 5 to 9 nm (D_TEM_V_) with temperature. At the same time, the silver phase underwent a more significant increase, especially for the sample prepared at 220 °C, which featured silver particles of about 30 nm with an overall larger size dispersity compared to the other samples (~ 24%). TEM images exhibit a gradual reduction in the number of separate ferrite NPs up to the point of obtaining only silver-ferrite heterostructures (sample Ag@Co8), in which 20 nm silver NPs are surrounded by 7 nm CoFe_2_O_4_ NPs. HRTEM images of the samples clearly show the architecture of the flower-like NHs (Fig. 4), also confirmed by the interlayer distances. Moreover, it is possible to observe the typical decahedral shape of silver oriented along its five-fold axis^56,57^.

The temperature of 200 °C (Ag@Co8) was therefore considered the best for maximizing the obtainment of the heterostructures and preserving low size dispersity.

As for the pair Ag@Co6/Ag@Co3, also the sample Ag@Co7 shows a slight decrease in the values of D_TEM_V_, accompanied by a negligible increase of the cobalt ferrite fraction if compared with the sample Ag@Co4. Contrarily, for Ag@Co8/Ag@Co5, significant changes in the particle sizes, as well as in the relative fraction of the two phases, were detected. In fact, Ag@Co5 (prepared at double concentration, 0.125 mmol in 10 mL total) features a larger size, higher presence of free cobalt ferrite NPs, and higher silver fraction. Moreover, at high concentration (10 mL of solvents in total) the supernatant, after the solvothermal treatment, featured dark color, due to the presence of smaller particles, mostly cobalt ferrite (73% w/w, Fig. 8S, Table 4S), that did not reach the critical size to deposit at the bottom of the Teflon liner. Conversely, at lower concentrations (20 mL of solvents in total), the supernatant was almost transparent. All these findings allowed us to conclude about the combined effect of temperature and dilution:

- In all the three cases, the dilution (by doubling the solvent) and the corresponding increase in the pressure led to the obtaining of more homogeneous silver-ferrite heterostructures.
- The combined effect of dilution and temperature was proved mainly when 20 mL of solvent in total and temperature of 200 °C were employed. Indeed, in this case, the fraction of cobalt ferrite increased due to the number of magnetic particles that covered the silver surface, limiting their growth.

### Effect of water

Another attempt was made starting from the sample Ag@Co8, prepared at 200 °C in 10 mL of pentanol and toluene, by adding 0.1 mL of water (Ag@Co10, Fig. 5), which correspond to 5.6 mmol, more than the double of the water already present in the solvents as an impurity. However, as hypothesized at the beginning, both XRD and TEM analyses reveal the obtainment of large silver particles (over 100 nm in size), surrounded by small ferrite NPs, which did not seem to be affected by the slight increase of the amount of water, contrary to silver. This is a confirmation of the detrimental effect of water on the silver NPs growth.

### Effect of chemical nature of the oleate

Finally, the same synthesis condition as for Ag@Co8 was tested by employing Fe^II^-oleate, instead of Co^II^-Fe^III^ oleate, to form silver-spinel iron oxide (magnetite/maghemite) heterostructures (Ag@Fe1, Table 1). As can be seen from XRD, TEM, and HRTEM analyses (Fig. 5, Fig. 9S) similar results were obtained in terms of particle size, heterostructure homogeneity, absence of free spinel ferrite NPs, and the relative amount of the phases. Therefore, the selected synthetic conditions, achieved after a series of attempted experiments, disclosed to be valid to prepare flower-like silver-spinel ferrite heterostructures also with different chemical nature of the magnetic part.

### Optical and magnetic properties

The sample Ag@Co8, featuring homogeneous flower-like heterostructures formed by a silver core and cobalt ferrite petals, was analyzed by several techniques to deepen the optical and magnetic properties (Fig. 6). The UV–Vis spectrum of Ag1 NPs used as seeds shows the presence of a sharp and well-defined plasmonic peak at about 410 nm (Fig. 6, upper left), in line with other silver NPs of the same size^10,36^. In the ferrite-silver heterostructure, the peak is broader and shifted towards higher wavelengths (around 430 nm). The red-shift could be due to the increased size of silver NPs (from 5 to 20 nm), even though in the literature a wide range of plasmonic absorption peak positions are reported for silver nanoparticles of about 15–20 nm (from 400 to 420 nm)^58–60^. Nevertheless, the broadening of the peak has not been observed for silver nanoparticles having different sizes^58^ even in systems with high size dispersity^61^. Since silver NPs are very sensitive to the refractive index of their surroundings^62^, spinel ferrites can induce optical changes in the heterostructures, that increase as the silver surface is covered by the ferrite and can cause the broadening of the plasmonic peak, as already observed in ferrite-silver heterostructures^10,25^.

Field (at 300 K and 10 K) and temperature (ZFC–FC protocols) dependences of the magnetization of the sample Ag@Co8 were measured (Fig. 6), to study the magnetic properties of the spinel ferrite component of the heterostructure, and compared with two cobalt ferrite samples, CoA and CoB, having slightly smaller and larger sizes respectively (Table 2), prepared by the oleate-based solvothermal method and described in previous work^6,35^.

The M *vs.* H curves of all samples recorded at 300 K show no hysteresis (Fig. 6, top right), typical for NPs in the superparamagnetic state, and are almost superimposable. Indeed, also the mean magnetic moment estimated from the curve using the software MINORIM (Fig. 10S) and the corresponding magnetic diameter (D_MAG_) calculated through Eq. 2 are similar for all samples (Table 2). At 10 K, the appearance of a large hysteresis occurred, characteristic of hard-magnetic behavior of NPs in the blocked state^6,38,64,65^. The coercive and anisotropy field, and the M_r_/M_s_ are lower for the silver-cobalt ferrite heterostructure, as well as the maximum, difference, and blocking temperatures estimated from the temperature-dependence of the magnetization (ZFC–FC, Fig. 6 upper right, Table 2). This is probably due to a decrease in the interparticle interactions in Ag@Co8 with respect to the bare cobalt ferrite NPs, as also indicated by the differently shaped FC curves. Indeed, the M_FC_ of the NHs tends to decrease more rapidly with increasing temperature in comparison to the other samples. From both the ZFC curve and distribution of blocking temperatures (Fig. 11S), it can be seen that no other peaks are visible, ascribable to other populations of magnetic nanoparticles, in agreement with TEM observation (Fig. 2c).

**Table 2:** Magnetic parameters of the samples: mean magnetic moment (μ_m_); magnetic diameter (D_MAG_) obtained by MINORIM;^63^ maximum (T_max_), difference (T_diff_), and blocking temperature (T_b_); coercive (H_c_^10^) and anisotropy field (H_K_^10^) calculated at 10 K; ratio between remanent and saturation magnetization (M_s_/M_r_) at 10 K. Mössbauer parameters obtained by NORMOS: type of the signal; isomer shift (δ); hyperfine field (B_hf_), full width at half maximum (fwhm).

Sample	µ_m_ (µ_B_∙10^3^)	D_MAG_ (nm)	D_XRD_ (nm)	D_TEM_V_ (nm)	T_max_ (K)	T_diff_ (K)	T_b_ (K)	H_c_^10^ (T)	H_K_^10^ (T)	M_r_/M_s_	Signal	δ (mm/s)	B_hf_ (T)	fwhm (mm/s)
Ag@Co8	2.13	4.2	11	7	173	198	117	1.00	3.4	0.51	Singlet	0.25	–	1.6
											Sextet	0.28	46.0	0.5
CoA	2.03	4.1	7	6	195	270	126	1.28	4.2	0.55	Singlet	0.20	–	12
											Singlet	0.44	–	1.1
CoB	2.51	4.4	10	8	241	266	163	1.32	3.8	0.62	Singlet	0.22	–	1.3
											Sextet	0.33	46.1	1.1

The magnetic and structural properties were also analyzed through ^57^Fe Mössbauer spectroscopy at room temperature (Fig. 7) since it allows us to study the magnetic spin with different timing (the Mössbauer measurement time window (10^–7^/10^–9^ s) is faster than the PPMS one (~ 1 s))^38,66,67^. The hyperfine parameters are reported in Table 2.

The cobalt ferrite sample CoA features two singlets: the first broad one associated with particles having a relaxation time near the Mössbauer measurement time window (τ_M_), while the second sharper one is related to particles in the superparamagnetic state. The samples Ag@Co8 and CoB, on the contrary, reveal the presence of one sextet and one singlet. While the singlet is analogous to the sharp one of CoA, the sextet derives from cobalt ferrite nanoparticles in the blocked state (having relaxation time above τ_M_). The hyperfine field and the isomer shift of the Ag@Co8 sextet are equal to 46.0 T and 0.28 mm/s, respectively (Table 2), values close those of CoB (B_hf_ = 46.1 T; δ = 0.33 mm/s). The difference between particles in the edge between superparamagnetic state and blocked state is indeed very narrow, a small difference in the diameter correspond to more significant differences in the volume, therefore the slightly smaller size of CoA (Table 2), if compared to CoB and Ag@Co8, results in the appearance of the broad singled instead of a sextet. It is interesting also to note the differences between magnetometry and Mössbauer measurements, probably ascribable to the different measurement time windows.

To sum up, although the important difference in terms of architecture between the bare magnetic nanoparticles (CoA and CoB), used as reference samples, and the Ag@Co8 NHs, the magnetic parameters are comparable. The slight differences can be ascribed both to the different sizes of the spinel ferrite petals in the NHs and the cobalt ferrite used for comparison (as evidenced by ^57^Fe Mössbauer spectroscopy), and the interaction that in the case of the NHs are softened by the silver cores.

## Conclusions

An oleate-based solvothermal method was employed for the first time for the synthesis of silver-spinel ferrite nanoheterostructures (NHs). FCC-silver NPs of about 7 nm, prepared through thermal reduction of silver oleate, features a high concentration of defects, mainly stacking faults along the {111} and {200} planes and twins. These types of defects, still present in silver nanoparticles of the heterostructures, should favor the growth of spinel ferrite NPs on their surface, generating flower-like shaped NHs with a silver core of about 20 nm and a dozen of spinel ferrite petals of about 7 nm. The method was optimized to maximize the production of NHs over the separate phases, tuning synthesis parameters such as the amount of water, the reaction temperature, the concentration of metal oleate, and the amount of organic solvents. Both cobalt ferrite and spinel iron oxide coupled with silver were successfully obtained by the same approach. The optical properties of the heterostructures confirmed the localized surface plasmon resonance effect and the comparison with the starting seeds allowed to evidence the significant influence of the magnetic part on its properties. The magnetic properties of the CoFe_2_O_4_-Ag NHs, studied by DC magnetometry and ^57^Fe Mössbauer spectroscopy, highlighted their hard-magnetic behavior, similar to that of cobalt ferrite NPs having a similar magnetic size, indicating no significant influence of the silver part.

## Experimental

### Chemicals

Oleic acid (90%) and iron chloride tetrahydrate (98%) were purchased from Alpha Aesar. 1-pentanol (≥ 99%), ethanol (99.8%), iron nitrate nonahydrate (≥ 98%), n-hexane (≥ 97%), silver nitrate (> 99%), sodium hydroxide (98%), and toluene (99.7%) were purchased from Sigma-Aldrich. Dimethyl sulfoxide (DMSO, ≥ 99.5%) wash purchased from Sigma.

### Methods

#### Synthesis of silver seeds

Silver NPs (Ag1) was synthesized following a reported procedure^36^. Firstly, silver oleate was prepared as follows. 10 mmol of NaOH were dissolved in 5 mL of distilled H_2_O, followed by the addition of 5 mL of ethanol, 10 mmol of oleic acid, and 25 mL of water. The so-prepared sodium oleate in hydroalcoholic solution (35 mL) was placed under vigorous stirring (700 rpm), and 50 mL of 0.2 M AgNO_3_ aqueous solution was slowly added. The white precipitate was filtered and dried at 30 °C for two days. Then, around 2 mmol of silver oleate was dissolved in 200 mL of oleic acid at 100 °C under nitrogen flux and vigorous stirring (700 rpm). After 15 min, the temperature was increased to 170 °C (heating rate 4 °C min-1) and allowed to react for 1 h, then cooled down to room temperature. The NPs were separated by centrifugation, washed three times with 5 mL of hexane and 5 mL of ethanol, and finally stored in hexane. The sample Ag2 was prepared following the same procedure reported in the following paragraph for the heterostructures, employing only 25 mg of oleate-capped Ag1 NPs and the solvents (10 mL of toluene, 10 mL of 1-pentanol), at 200 °C for 10 h.

#### Synthesis of silver-spinel ferrite heterostructures

Ag1 NPs were used as seeds to prepare silver-spinel ferrite heterostructures. Around 25 mg of oleate-capped Ag1 NPs were dispersed in toluene and placed into a Teflon liner together with the desired amount of metal oleate (synthesized as described in previous works)^30,35,49^, 1-pentanol and water, as reported in Table 1. The liner was then enclosed in a stainless-steel autoclave (Berghof DAB-2), briefly shaken and put vertically into a pre-heated oven (140–220 °C) for 10 h. After the treatment, the particles were separated with a magnet and the supernatant discarded. The particles were dispersed in 10 mL of hexane, then sedimented by adding 10 mL of ethanol. The washing procedure was repeated twice. Finally, the products were stored in 5 mL of hexane.

### Characterization

The samples were characterized by X-ray Diffraction (XRD) using a Seifert X3000 Cu Kα radiation (1.5418 Å). Calibration of peak position and instrumental width was done using powder LaB_6_ from NIST. The refinement of the structural parameters^68^ was performed by the Rietveld method using the MAUD software^69^, adopting the recommended fitting procedures^70^. CIF structure COD IDs used for the refinement are 9008459, 1509145, 1533163, and 1010369 for silver fcc, silver hcp, cobalt ferrite, and spinel iron oxide, respectively.

TEM images were obtained by using a JEOL JEM 1400 Plus operating at 120 kV. The particle size distribution was obtained by measuring over 1000 particles with the aid of the software Pebbles, setting spherical shape for the elaboration^71^. The mean particle diameter was calculated as the average value and the dispersity as the percentage ratio between the standard deviation and the average value. The volume-weighted particle diameter was calculated as:

1 $$D_{TEMV} = D_{TEM} e^{{\left( {3\sigma^{2} } \right)}}$$

where σ is the percentage standard deviation.

HRTEM images were carried out using JEOL JEM 2010 UHR equipped with a Gatan 794 slow-scan CCD camera. The images were analyzed through the software Digital Micrograph, to obtain the Fast Fourier Transform (FFT) and the inversed-FFT. The geometric phase analysis (GPA)^72^ was obtained with STEM_CELL^51,52^.

Fourier Transform Infrared (FT-IR) spectra were recorded in the region from 400 to 4000 cm^−1^ by using a Bruker Equinox 55 spectrophotometer. Samples were measured in a KBr pellet. Spectra have been processed using OPUS software.

Thermogravimetric analyses (TGA) curves were obtained by using a PerkinElmer STA 6000, in the 25–850 °C range, with a heating rate of 10 °C min^−1^ under 40 mL min^−1^ O_2_ flow.

Room temperature ^57^Fe Mössbauer spectroscopy was performed on a Wissel spectrometer using transmission arrangement and proportional detector LND-45431. An α-Fe foil was used as a standard, and the fitting procedure was done by NORMOS program to determine the isomer shift, quadrupole splitting, hyperfine field, and full width at half maximum of the signals.

DC magnetic properties were studied by means of a Quantum Design PPMS Dynacool (H_max_ = 90 kOe) by using the VSM module. Different kinds of magnetic measurements were carried out. The field dependence of the magnetization (M *vs.* H) was studied at 10 K and 300 K between 7 T and -7 T. The magnetic domain size (D_MAG_) distribution was estimated according to the following equation:

2 $$D_{MAG} = \sqrt[3]{{\frac{{6\mu a^{3} }}{{\mu_{uc} \pi }}}}$$

where a is the lattice parameter, μ_uc_ the magnetic moment of the unit cell of the spinel ferrite (33.6 μ_B_), μ_0_ the mean magnetic moment, calculated from the field-dependent magnetization curve recorded at 300 K by means of MINORIM software^63^, which uses a non-regularized method. The temperature dependence of the magnetization (M *vs*. T) was studied by using the Zero-Field-Cooled (ZFC) and Field-Cooled (FC) protocols: the sample was cooled down from 300 to 5 K in a zero magnetic field; then, the signals were recorded under a static magnetic field of 10 mT. M_ZFC_ was measured during the warm-up from 5 to 380 K, whereas M_FC_ was recorded during the cooling step.

UV–Visible spectra were acquired on a Cary 50 Probe using a Xenon lamp. The samples were diluted with hexane and analyzed on a quartz cuvette.

## Supplementary information

Supplementary Information.
