# Supplementary material for: Defect-assisted synthesis of magneto-plasmonic silver-spinel ferrite heterostructures in a flower-like architecture
Source: Sci Rep. 2020 Oct 12;10:17015. doi: 10.1038/s41598-020-73502-5 (PMC7550332; doi:10.1038/s41598-020-73502-5)
Supplement: Supplementary file 1 — Supplementary Information. [file 41598_2020_73502_MOESM1_ESM.docx]

Supplementary Material for

**Defect-Assisted Synthesis of Magneto-Plasmonic Silver-Spinel Ferrite Heterostructures in a Flower-Like Architecture**.

Marco Sanna Angotzi, Valentina Mameli, Claudio Cara, Vincenzo Grillo, Stefano Enzo, Anna Musinu, Carla Cannas

Table 1S. Silver-spinel ferrite NHs reported in the literature.

| **System** | **Architecture** | **Synthesis** | **Seed** | **Silver Precursor** | **Spinel Ferrite Precursor** | **Solvent** | **C Ag (mM)** | **T (°C)** | **Ref** |
| --- | --- | --- | --- | --- | --- | --- | --- | --- | --- |
| Ag@Fe_3_O_4_ | Dimer | Thermal Decomposition | Ag | AgNO_3_ | Fe Oleate | 1-octadecene | 20 | 310 | ^1^ |
| Ag@Fe_3_O_4_ | Multimer | Thermal Decomposition | Ag | Ag(PPh_3_)_2_NO_3_ | AgNO_3_ | benzyl ether | 40 | 300 | ^2^ |
| Ag@Fe_3_O_4_ | Dimer | Thermal Decomposition | Ag | AgNO_3_ | AgNO_3_ | benzyl ether | - | 290 | ^3^ |
| Ag@Fe_3_O_4_ | Dimer | Thermal Decomposition | Ag | Ag(ac) | Fe(acac)_3_ | 1-octadecene | 200 | 260 | ^4^ |
| Ag@Fe_3_O_4_ | Flower-like | Thermal Decomposition | Ag | Ag(ac) | Fe(acac)_3_ | Diphenyl Ether | 200 | 260 |  |
| Ag@Fe_3_O_4_ | Core-shell | Thermal Decomposition | Ag | Ag(acac) | Fe(acac)_3_ | Diphenyl Ether | 28 | 260 | ^5^ |
| Fe_3_O_4_@Ag | Multimer | Surface Reduction | Fe_3_O_4_ | Ag Oleate | Fe Oleate | 4-tert-butyltoluene | 12 | 100 | ^6^ |
| Fe_3_O_4_@Ag | Multimer | Surface Reduction | Fe_3_O_4_ | Ag Oleate | Fe Oleate | Toluene | 12 | 100 |  |
| Fe_3_O_4_@Ag | Dimer | Surface Reduction | Fe_3_O_4_ | Ag Oleate | Fe Oleate | Diphenyl Ether | 12 | 100 |  |
| Fe_3_O_4_@Ag | Dimer | Surface Reduction | Fe_3_O_4_ | Ag Oleate | Fe Oleate | dichlorobenzene | 12 | 100 |  |
| CoFe_2_O_4_@Ag | Flower-like | Surface Reduction | CoFe_2_O_4_ | AgNO_3_ | FeCl_2_ | Water | >0.01 | 70 | ^7^ |
| Fe_3_O_4_@Ag | Dimer | Surface Reduction | Fe_3_O_4_ | AgNO_3_ | Fe(acac)_3_ | Hexane:Water 1:2 | 0.004 | RT | ^8^ |
| Fe_3_O_4_@Ag | Dimer | Surface Reduction | Fe_3_O_4_ | AgNO_3_ | Fe(CO)_5_ | Water | 0.1 | 60 | ^9^ |
| Ag@Fe_3_O_4_ | Dimer | Surface Reduction | Fe_3_O_4_ | Ag(ac) | Fe Oleate | Toluene | 7 | 80 | ^10^ |
| Ag@Fe_3_O_4_ | Dimer | Surface Reduction | Fe_3_O_4_ | [Ag(NH_3_)_2_]^+^ | FeCl_2_-FeCl_3_ | Water | 30 | 70 | ^11^ |
| Ag@Fe_3_O_4_ | Dimer | Surface Reduction | Fe_3_O_4_ | AgNO_3_ | Fe Oleate | Water | 100 | RT | ^12^ |
| Ag@Fe_3_O_4_ | Dimer | Surface Oxidation | Fe | AgNO_3_ | Fe(CO)_5_ | 1- octadecene:  Toluene 4:1 | 0.006 | 205 | ^13^ |
| Ag@Fe_3_O_4_ | Dimer | Surface Oxidation | Fe | AgNO_3_ | Fe(CO)_5_ | 1-octadecene:  Oleilamine 4:1 | 8 | 120 | ^13^ |
| Ag@Fe_3_O_4_ | Dimer/  Flower-like | 1-Pot Thermal Decomposition | - | Ag(ac) | Fe(acac)_3_ | 1- octadecene:  Diphenyl Ether | 17 | 240 | ^14^ |
| Ag@Fe_3_O_4_ | Dimer/  Flower-like | 1-Pot Hydrothermal | - | AgNO_3_ | NH_4_Fe_2_(SO_4_)_2_ | Water | 25 | 160 | ^15^ |
| Ag@Fe_3_O_4_ | Flower-like | 1-Pot Hydrothermal | - | AgNO_3_ | FeCl_2_ | Water | 30-700 | 125 | ^16^ |
| Ag@Fe_3_O_4_ | Dimer/  Flower-like | 1-Pot Solvothermal | - | Ag Oleate | Fe Oleate | 1-octadecene | 8 | 150 | ^17^ |
| Ag@CoFe_2_O_4_ | Flower-like | Solvothermal | Ag | Ag Oleate | Fe Oleate | Toluene:  Pentanol 1:1 | 10 | 200 | This work |
| Ag@Fe_3_O_4_ | Flower-like | Solvothermal | Ag | Ag Oleate | Fe Oleate | Toluene:  Pentanol 1:1 | 10 | 200 | This work |

**SILVER NPs ANALYSIS**

Figure 1S. FTIR spectra (left side) of silver oleate and silver NPs; TGA curve (right side) of silver NPs.


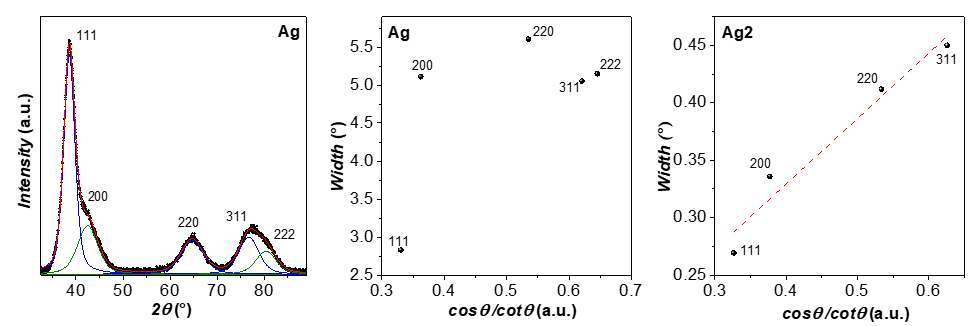


Figure 2S. Fitting of Ag XRD pattern with pseudo-Voight functions (left side); peak width evolution with the angle for Ag (middle) and Ag2 (right side) samples.

Table 2S. XRD parameters for Ag sample extracted from single peak analysis by fitting with pseudo-Voight function: Miller’s indexes of the reflection, position (2θ), width (FWHM), size estimated by Scherrer equation (D), lattice parameter (a), and j<G>, obtained from reference,^18^ which is an indication of the intensity and direction of the peak shift.

| **Reflex** | **2θ (°)** | **FWHM (°)** | **D (nm)** | **a (Å)** | **j<G>** |
| --- | --- | --- | --- | --- | --- |
| 111 | 38.6 | 2.3 | 3.6 | 4.04 | +3.95 |
| 200 | 42.5 | 5.1 | 2.0 | 4.26 | -7.90 |
| 220 | 64.7 | 5.6 | 2.1 | 4.08 | +3.95 |
| 311 | 76.7 | 5.1 | 2.5 | 4.12 | -1.44 |
| 222 | 80.4 | 5.1 | 2.5 | 4.14 | -1.98 |


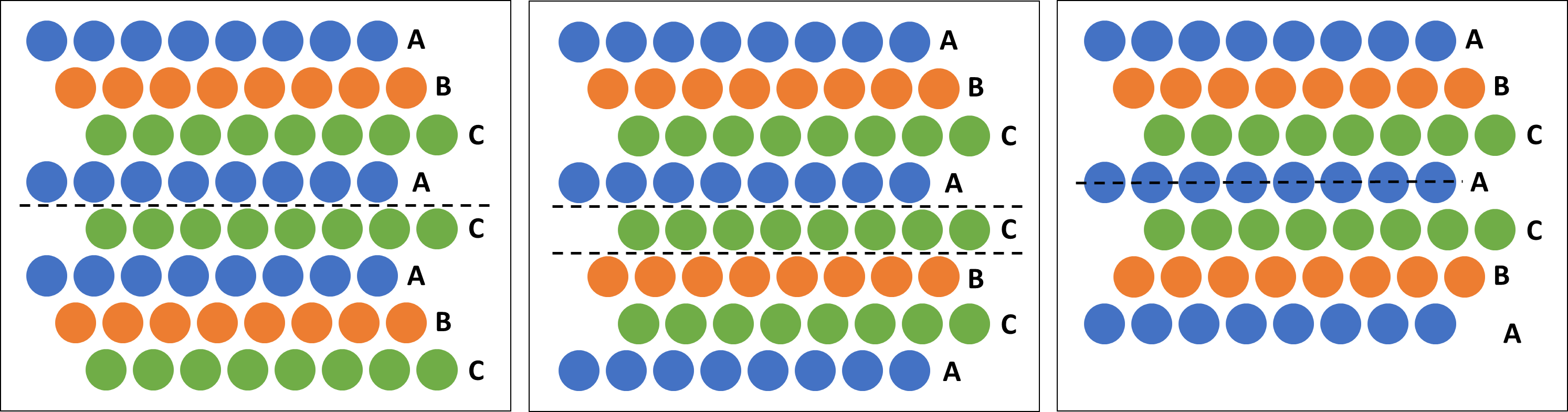


Figure 3S. Intrinsic (left), extrinsic (middle), and twin (right) stacking faults in a fcc structure.

Figure 4S. Rietveld refinements obtained by MAUD^19^ of XRD patterns of Ag by using fcc structure (a), both fcc and hcp structures (b), and only fcc structure with Warren planar defects model correction (c). The broad band at about 20° is due to the glass sample holder.

Table 3S. Rietveld refinement parameters obtained by MAUD^19^ for some selected samples: cell parameters (a, c); microstrains (<ε>); intrinsic (α’), extrinsic (α’’), and twin (β) stacking faults.

| **Sample** | **R_WP_** | **Structure** | **Fraction (% w/w)** | **a (Å)** | **c (Å)** | **D (nm)** | **<ε>** | **Warren α’** | **Warren α’’** | **Warren β** |
| --- | --- | --- | --- | --- | --- | --- | --- | --- | --- | --- |
| Ag | 9.3 | Silver fcc | 100 | 4.165 | - | 5 | 3·10^-2^ | - | - | - |
| Ag | 5.4 | Silver fcc | 55 | 4.082 | - | 8 | 3·10^-2^ | - | - | - |
|  |  | Silver hcp | 45 | 2.988 | 4.689 | 55 | 5·10^-2^ | - | - | - |
| Ag | 3.6 | Silver fcc | 100 | 4.145 | - | 14 | 1·10^-2^ | 0.14 | 7·10^-3^ | 4·10^-4^ |
| Ag2 | 20.0 | Silver fcc | 100 | 4.084 | - | 44 | 2·10^-7^ | - | - | - |
| Ag2 | 20.0 | Silver fcc | 100 | 4.084 | - | 44 | 8·10^-7^ | 3·10^-11^ | 2·10^-5^ | 2·10^-9^ |
| Ag@Co1 | 3.9 | Silver fcc | 14 | 4.089 | - | 9 | 2·10^-3^ | - | - | - |
|  |  | Cobalt Ferrite | 86 | 8.411 | - | 7 | 1·10^-2^ | - | - | - |
| Ag@Co1 | 3.1 | Silver fcc | 24 | 4.089 | - | 19 | 2·10^-3^ | 2·10^-2^ | 5·10^-3^ | 4·10^-3^ |
|  |  | Cobalt Ferrite | 76 | 8.413 | - | 7 | 1·10^-6^ | - | - | - |
| Ag@Co8 | 3.8 | Silver fcc | 67 | 4.093 | - | 9 | 8·10^-5^ | - | - | - |
|  |  | Cobalt Ferrite | 33 | 8.428 | - | 13 | 6·10^-3^ | - | - | - |
| Ag@Co8 | 1.6 | Silver fcc | 66 | 4.091 | - | 29 | 1·10^-5^ | 2·10^-2^ | 1·10^-5^ | 3·10^-2^ |
|  |  | Cobalt Ferrite | 34 | 8.418 | - | 11 | 6·10^-3^ | - | - | - |


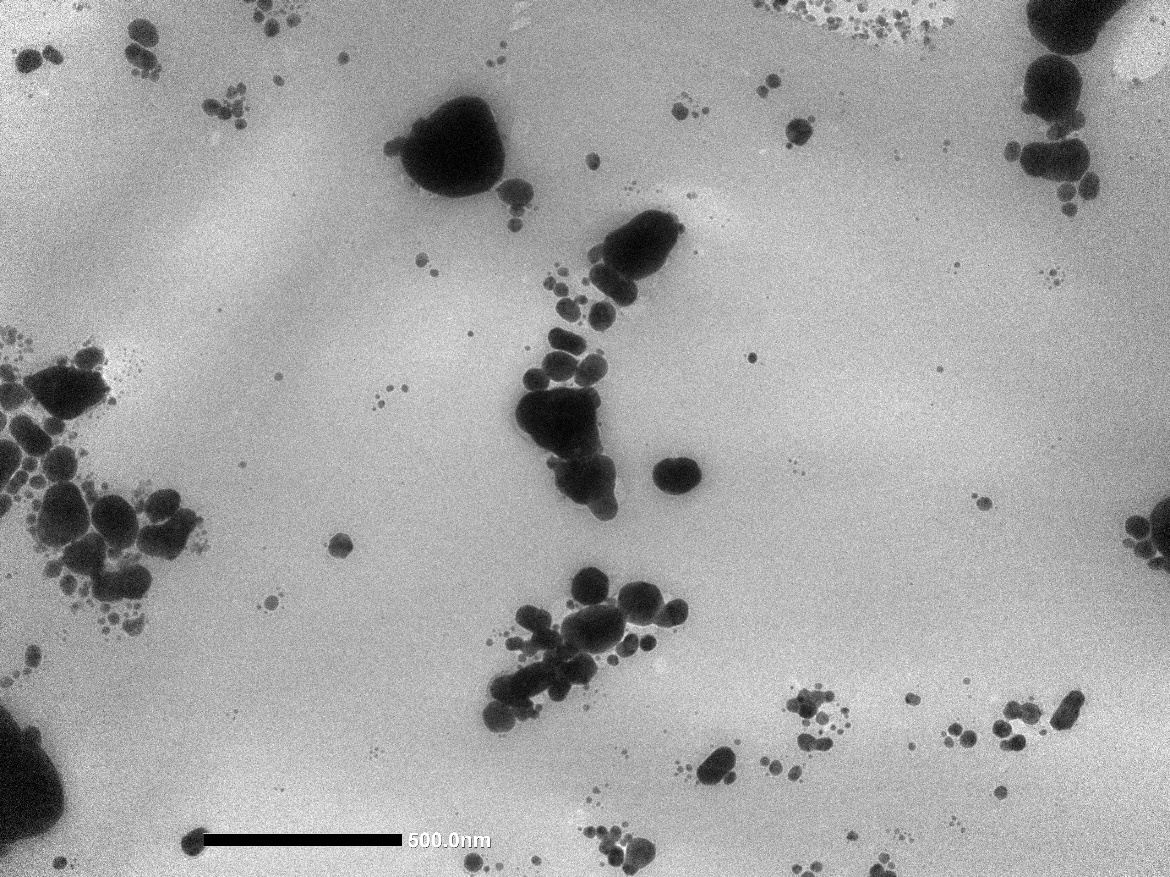


Figure 5S. Rietveld refinements obtained by MAUD^19^ of XRD patterns (left) of Ag2 by using fcc structure (a) and fcc structure with Warren planar defects model correction (b); TEM image of Ag2 (right).

**HETEROSTRUCTURES ANALYSIS**

Figure 6S. Rietveld refinements obtained by MAUD^19^ of XRD patterns of Ag@Co1 (left) and Ag@Co8 (right) by using fcc structure (a) and fcc structure with Warren planar defects model correction (b). The broad band at about 20° is due to the glass sample holder.

**
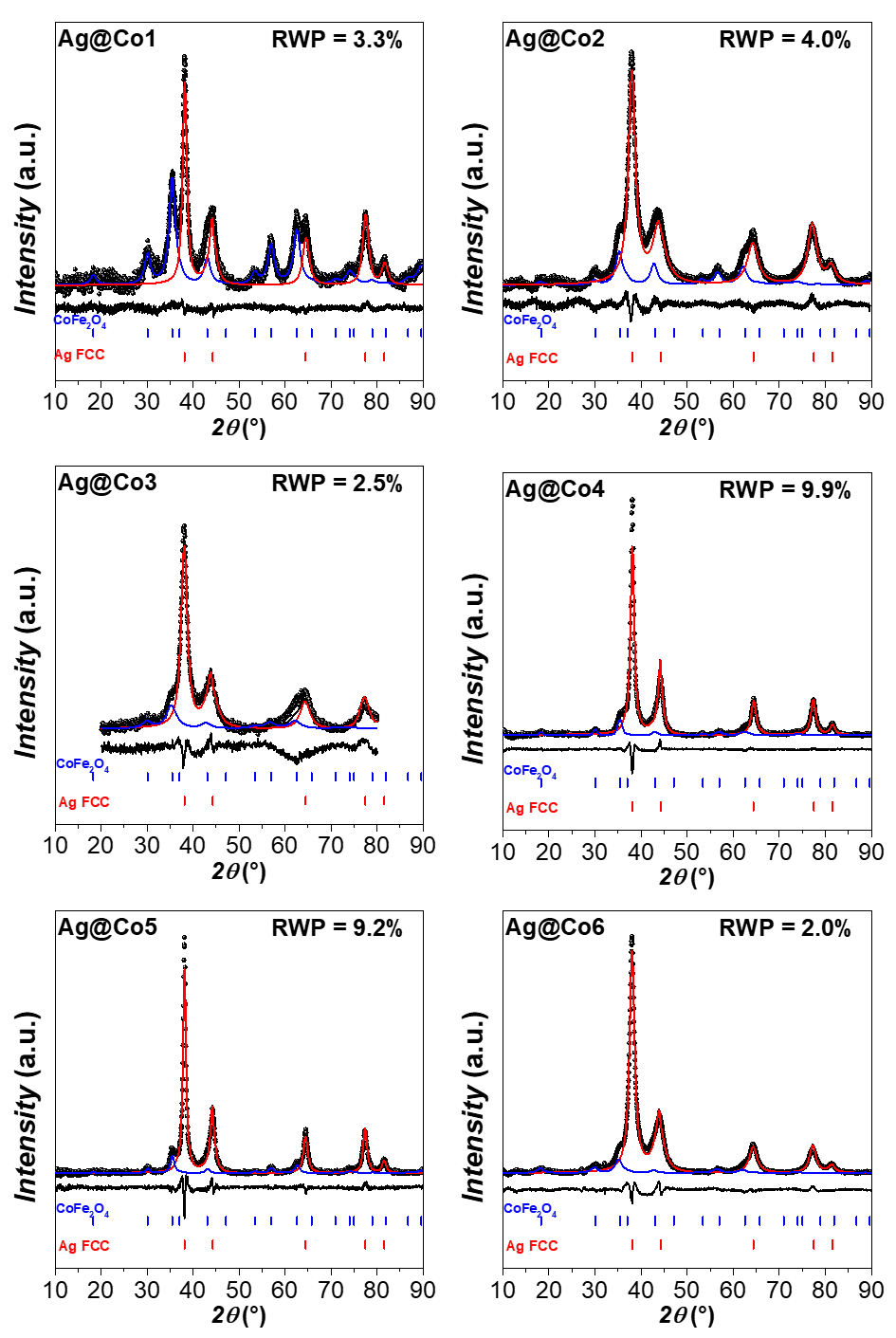
**
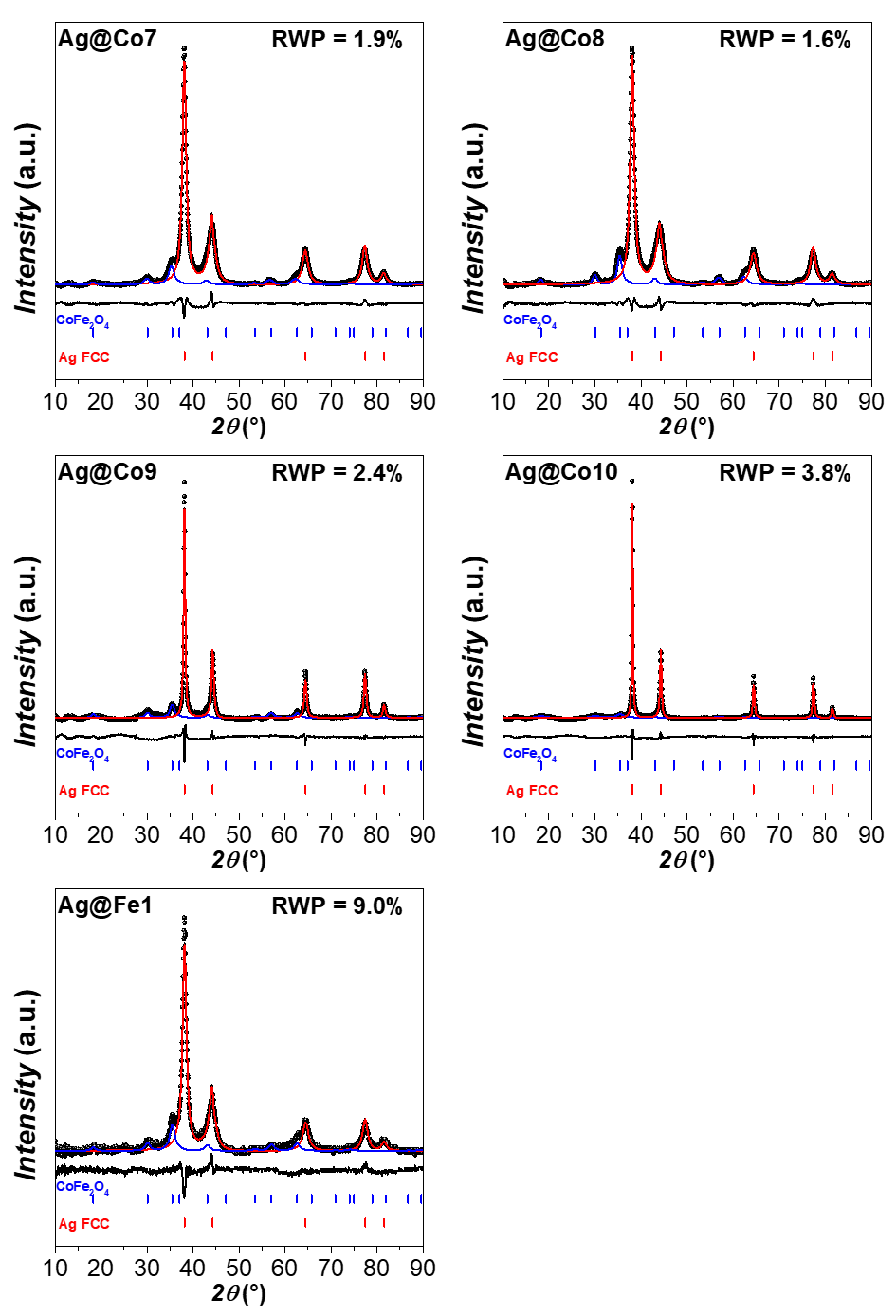


Figure 7S. Rietveld refinement obtained by MAUD^19^ of the XRD patterns of the silver-spinel ferrite heterostructures.


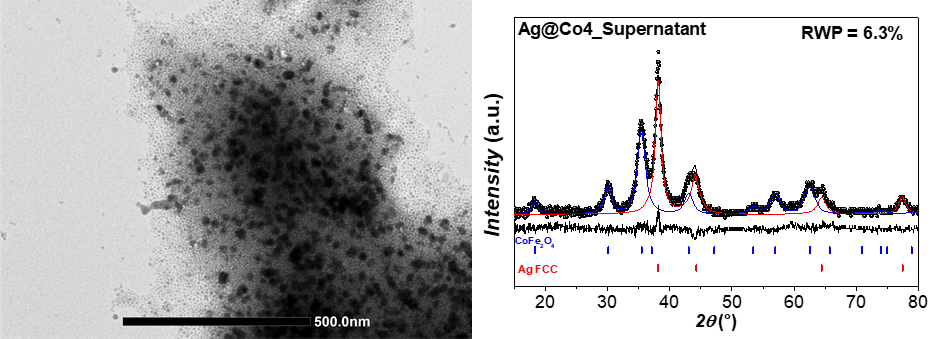


Figure 8S. TEM image (left) and Rietveld refinement obtained by MAUD^19^ (right) of the supernatant mixture from the synthesis of Ag@Co5.

Table 4S. Crystallite size, cell parameter, quantitative analyses calculated by Rietveld refinement, and particle size calculated from TEM images.

| **Sample** | **Phase** | **Microstrain** | **a (Å)** | **Fraction (% w/w)** | **<D_XRD_> (nm)** | **<D_TEM_V_> (nm)** |
| --- | --- | --- | --- | --- | --- | --- |
| Ag@Co5 | CoFe_2_O_4_ | 4∙10^-3^ | 8.390 | 24 | 12(1) | 10(2) |
|  | Ag FCC | 1∙10^-4^ | 4.089 | 76 | 28(2) | 31(6) |
| Ag@Co5_supernatant | CoFe_2_O_4_ | 7∙10^-3^ | 8.404(2) | 73 | 9(1) | 4(1) |
|  | Ag FCC | 2∙10^-5^ | 4.088(2) | 27 | 20(2) | 24(4) |


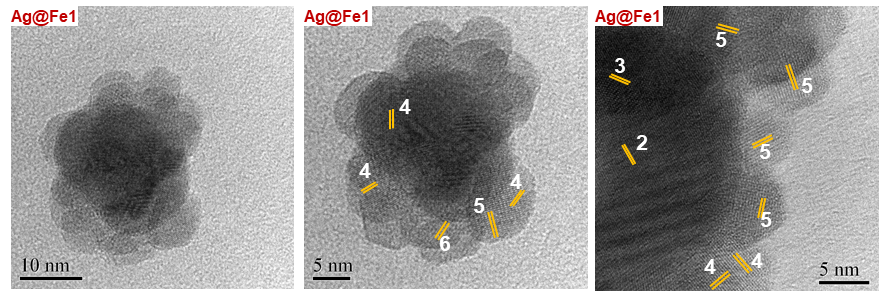


Figure 9S. HRTEM images of Ag@Fe1 obtained by Digital Micrograph.

**DC MAGNETOMETRY**

Figure 10S. Field-dependent magnetization refinement of Ag@Co8 sample by using MINORIM.^20^ In the insets, the resulting magnetic moment distributions.

Figure 11S. Anisotropy energy barrier distributions estimated by the first derivative *-d(M_FC_-M_ZFC_)/dT* and particle size distribution of the samples CoA, CoB, and Ag@Co8.

**REFERENCES**

1 F. Lin, W. Chen, Y.-H. Liao, R. Doong and Y. Li, *Nano Res.*, 2011, **4**, 1223–1232.

2 O. Moscoso-Lodoño, D. Muraca, P. Tancredi, C. Cosio-Castañeda, K. R. Pirota and L. M. Socolovsky, *J. Phys. Chem. C*, 2014, **118**, 13168–13176.

3 M. E. F. Brollo, R. López-Ruiz, D. Muraca, S. J. A. Figueroa, K. R. Pirota and M. Knobel, *Sci. Rep.*, 2015, **4**, 6839.

4 P. Tancredi, O. Moscoso Londoño, P. C. Rivas Rojas, U. Wolff, L. M. Socolovsky, M. Knobel and D. Muraca, *J. Phys. D. Appl. Phys.*, , DOI:10.1088/1361-6463/aaccc3.

5 C.-H. Lai, T.-F. Wu and M.-D. Lan, *IEEE Trans. Magn.*, 2005, **41**, 3397–3399.

6 Y. Mao, P. Yi, Z. Deng and J. Ge, *CrystEngComm*, 2013, **15**, 3575.

7 M. Kaloti and A. Kumar, *J. Phys. Chem. C*, 2016, **120**, 17627–17644.

8 H. Gu, Z. Yang, J. Gao, C. K. Chang and B. Xu, *J. Am. Chem. Soc.*, 2005, **127**, 34–35.

9 J. Zeng, M. Gong, D. Wang, M. Li, W. Xu, Z. Li, S. Li, D. Zhang, Z. Yan and Y. Yin, *Nano Lett.*, 2019, **19**, 3011–3018.

10 M. Sahu, M. Shaikh, A. Rai and K. V. S. Ranganath, *J. Inorg. Organomet. Polym. Mater.*, 2020, **30**, 1002–1007.

11 C. Yong, X. Chen, Q. Xiang, Q. Li and X. Xing, *Bioact. Mater.*, 2018, **3**, 80–86.

12 Q. Ding, D. Liu, D. Guo, F. Yang, X. Pang, R. Che, N. Zhou, J. Xie, J. Sun, Z. Huang and N. Gu, *Biomaterials*, 2017, **124**, 35–46.

13 L. Pan, J. Tang and Y. Chen, *Sci. China Chem.*, 2013, **56**, 362–369.

14 J. Huang, Y. Sun, S. Huang, K. Yu, Q. Zhao, F. Peng, H. Yu, H. Wang and J. Yang, *J. Mater. Chem.*, 2011, **21**, 17930.

15 L. . b Pan, Y. . b Chen and F. . Wang, *Mater. Chem. Phys.*, 2012, **134**, 177–182.

16 A. Amarjargal, L. D. Tijing, I. T. Im and C. S. Kim, *Chem. Eng. J.*, 2013, **226**, 243–254.

17 Y. Shan, Y. Yang, Y. Cao and Z. Huang, *RSC Adv.*, 2015, **5**, 102610–102618.

18 C. N. J. Wagner, *Acta Metall.*, 1957, **5**, 427–434.

19 L. Lutterotti and P. Scardi, *J. Appl. Crystallogr.*, 1990, **23**, 246–252.

20 J. Van Rijssel, B. W. M. Kuipers and B. H. Erné, *J. Magn. Magn. Mater.*, 2014, **353**, 110–115.
